# Computational Identification of Phospho-Tyrosine Sub-Networks Related to Acanthocyte Generation in Neuroacanthocytosis

**DOI:** 10.1371/journal.pone.0031015

**Published:** 2012-02-15

**Authors:** Lucia De Franceschi, Giovanni Scardoni, Carlo Tomelleri, Adrian Danek, Ruth H. Walker, Hans H. Jung, Benedikt Bader, Sara Mazzucco, Maria Teresa Dotti, Angela Siciliano, Antonella Pantaleo, Carlo Laudanna

**Affiliations:** 1 Department of Medicine, University of Verona, Verona, Italy; 2 Department of Pathology and Diagnosis, University of Verona, Verona, Italy; 3 The Center for Biomedical Computing, University of Verona, Verona, Italy; 4 Neurologische Klinik und Poliklinik, Klinicum der Universität München, Munich, Germany; 5 Department of Neurology, James J. Peters Veterans Affairs Medical Center, Bronx, New York, New York, United States of America; 6 Department of Neurology, Mount Sinai School of Medicine, New York, New York, United States of America; 7 Department of Neurology, University Hospital Zurich, Zurich, Switzerland; 8 Department of Neurosciences, University of Verona, Verona, Italy; 9 Department of Neurology, University of Siena, Siena, Italy; 10 Department of Biology, Molecular and Medical Chemistry, University of Torino, Torino, Italy; Tokyo Medical and Dental University, Japan

## Abstract

Acanthocytes, abnormal thorny red blood cells (RBC), are one of the biological hallmarks of neuroacanthocytosis syndromes (NA), a group of rare hereditary neurodegenerative disorders. Since RBCs are easily accessible, the study of acanthocytes in NA may provide insights into potential mechanisms of neurodegeneration. Previous studies have shown that changes in RBC membrane protein phosphorylation state affect RBC membrane mechanical stability and morphology. Here, we coupled tyrosine-phosphoproteomic analysis to topological network analysis. We aimed to predict signaling sub-networks possibly involved in the generation of acanthocytes in patients affected by the two core NA disorders, namely McLeod syndrome (MLS, *XK*-related, Xk protein) and chorea-acanthocytosis (ChAc, *VPS13A*-related, chorein protein). The experimentally determined phosphoproteomic data-sets allowed us to relate the subsequent network analysis to the pathogenetic background. To reduce the network complexity, we combined several algorithms of topological network analysis including cluster determination by shortest path analysis, protein categorization based on centrality indexes, along with annotation-based node filtering. We first identified *XK-* and *VPS13A*-related protein-protein interaction networks by identifying all the interactomic shortest paths linking Xk and chorein to the corresponding set of proteins whose tyrosine phosphorylation was altered in patients. These networks include the most likely paths of functional influence of Xk and chorein on phosphorylated proteins. We further refined the analysis by extracting restricted sets of highly interacting signaling proteins representing a common molecular background bridging the generation of acanthocytes in MLS and ChAc. The final analysis pointed to a novel, very restricted, signaling module of 14 highly interconnected kinases, whose alteration is possibly involved in generation of acanthocytes in MLS and ChAc.

## Introduction

Acanthocytes, abnormal thorny red cells in the peripheral circulation, are one of the biological hallmarks of a severe and underrecognised group of neurodegenerative group of disorders known as the neuroacanthocytosis syndromes (NA). Genetic studies in the two core NA disorders, McLeod syndrome (MLS) and chorea-acanthocytosis (ChAc), have resulted in the identification of mutations on (i) the *XK* gene (X-chromosome) encoding for Xk protein in MLS and (ii) the *VPS13A* gene (chromosome 9), encoding for chorein in ChAc [1], [2], [3], [4], [5]. These two disorders share a similar Huntington disease-like phenotype including chorea, psychiatric and cognitive abnormalities and additional neuromuscular involvement. The gap between genotype and phenotype in both disorders suggests an important role for post-translational protein modifications, such as phosphorylation, in abnormal cell functions. Since red cells are easily accessible and acanthocytes are part of the clinical presentation of NA, the study of acanthocytes in NA represents a convenient experimental model to be exploited.

Red cells are characterized by a typical biconcave shape, which is maintained through their 120 day life in the peripheral circulation. The red cell membrane is formed by a lipid bilayer bound to integral proteins, such as band 3, and connected with the spectrin-actin cytoskeleton network by multicomplex proteins bridging the membrane to the cytoskeleton. Quantitative or qualitative changes in protein membrane composition result in abnormal red cell morphology [6]. Although molecular and functional studies in hereditary red cell membrane disorders such as hereditary spherocytosis (HS) or hereditary ovalocytosis (HOS) have highlighted the functional role of many of the membrane proteins, much still remains to be investigated in diseases characterized by abnormal red cell morphology and normal membrane protein composition. Recent studies have suggested a possible role of post-translational modifications, such as phosphorylation, in modulation of red cell membrane protein-protein function and/or structure. In addition, changes in the membrane protein phosphorylation state might result in loss of red cell membrane mechanical stability and abnormal morphology [7], [8], [9], [10], [11].

We applied methods of static network analysis derived from graph theory with the aim of identifying topological properties of signaling networks important in abnormal red cells from MLS and ChAc patients. We combined several algorithms of topological network analysis of the experimental data. In order to filter the network complexity, we combined cluster extraction and centrality analysis, coupled to multidimensional network node categorization. We extracted very restricted sub-networks with 14 highly interconnected kinases possibly involved in generation of acanthocytes in NA disorders.

## Results

### Sub network reconstruction and topological analysis

We performed comparative proteomic analysis combined with the identification of differently tyrosine phosphorylated proteins from red cell membranes of healthy and either ChAc or MLS subjects (Table 1). We observed no relation between the differently tyrosine phosphorylated proteins from red cells of either ChAc or MLS subjects and disease progression. Since the tyrosine phosphoproteomic analysis generated a significant amount of data, whose interpretation required a network level data analysis (Supplementary Table S1, S2, S3), we carried out a topological network analysis to identify potential new signaling pathways involved in generation of acanthocytes common to MLS and ChAc.

**Table 1:** Demographic and molecular data of control subjects and McLeod Syndrome and chorea-acanthocytosis patients.

	Gender	Age of symptom onset (yrs)	Age at examination (yrs)	Abnormalities on Clinical Examination	Molecular Defect	Ref.
Healthy Controls	3M/9F	-	35.6±2.3	-	-	[34]
ChAc 1	F	16	35	Orofacial dyskinesias, tics, chorea, tongue protrusion dystonia, dysarthria, absent reflexes	Splice site mutation intron 3 (c.188-5T>G); mutation on the other allele unknown	[34], [38]
ChAc 2	M	47	49	Tongue and lip biting, dysarthria, orofacial dyskinesias, steppage gait, no cognitive impairment	Splice site mutations in exon 58 (c.8105+3_+6del)and exon 70 (c.9275G>A; Chorein absent	[34], [42], [43]
ChAc 3	F	28	30	Seizures, orofacial dyskinesias, chorea, tongue protrusion dystonia, dysarthria, obsessive compulsive disorder, absent reflexes, neuropathy	p.A1428P and splice site mutation intron 55 (c.7806G>A); Chorein absent	[34], [39]
ChAc 4	F	24	40	Seizures, orofacial dyskinesias, chorea, tongue protrusion dystonia, absent reflexes, myopathy, neuropathy	p.R1297X and p.V1406CfsX20	[5], [34]
ChAc 5	M	16	47	Orofacial dyskinesias, dysarthria, chorea, psychiatric disorder, no seizures and no parkinsonism at age 30, neuropathy	Splice site mutations intron 22 (c.2288+2T>C) and intron 61 (c.8472-1G>C); Chorein absent	[5], [34], [38]
ChAc 6	F	32	56	Seizures, orofacial dyskinesias, chorea, tongue protrusion dystonia, dysarthria, dysphagia, absent reflexes, myopathy	Homozygous p.K372SfsX2	[11], [34], [40], [41]
ChAc 7	M	24	32	Seizures, orofacial dyskinesias, chorea, tongue protrusion dystonia, dysarthria, parkinsonism, absent reflexes, neuropathy, myopathy	Chorein absent	[34]
ChAc 8	F	24	38	Seizures, orofacial dyskinesias, chorea, tongue protrusion dystonia, psychiatric disorder	Splice site mutation intron 6 (c.495+5G>A) and p.K1635VfsX6	[34]
ChAc 9	F	28	40	Seizures, Orofacial dyskinesias, chorea, dysarthria, dysphagia, absent reflexes, myopathy	Chorein absent	[34]
MLS 1	M	58	60	Yawning, belching, dystonia, no seizures, mild facial masking (no bradykinesia, tremor, or hypertonia)	p.R222G	[36]
MLS 2	M	56	60	Gait problems, tongue-biting, dystonia, atrial fibrillation, anxiety, depression, no seizures, mild truncal chorea, hyporeflexia, myopathy, neuropathy	Deletion of exons 1 and 2	[37]
MLS 3	M	26	54	Bipolar disorder, schizophrenia, moderate perioral dyskinesias, pronounced generalized chorea, mild generalized muscular atrophy, absent reflexes, cardiopathy,	p.Q299X	[35]
MLS 4	M	25	41	Personality disorder, mild generalized chorea, absent reflexes	p.Q299X	[35]
MLS 5	M	20	47	Swallowing difficulties, gait problems, pronounced generalized chorea, tongue protrusion dystonia, feeding dystonia, head dropping, mild cognitive impairment generalized muscular atrophy, absent reflexes	p.Q299X	[35]

M; male; F: female; yrs: years; ChAc: chorea-acanthocytosis; MLS: Mcleod Syndrome; Control age is presented as means ± SD. Molecular defect” refers to the *VPS13A* and *XK* gene, respectively, that are responsible for ChAc and MLS.

The first step of our analysis was intended to demonstrate signaling mechanisms possibly linking Xk and chorein protein to the specific pattern of protein tyrosine phosphorylation observed in red cells isolated from MLS and ChAc patients. We first reconstructed Xk and chorein-related networks by identifying all the interactomic shortest paths linking Xk and chorein to the corresponding set of proteins whose phosphorylations were altered in patients. In these sub-networks, we looked for a possible connection between defective Xk or chorein and the experimentally determined tyrosine phosphoproteomic patterns observed in red cells (see Methods). The Xk_to_P-tyr-network consisted of 129 proteins and 738 interactions (Appendix S1 and S2). The chorein_to_P-tyr-network consisted of 132 proteins and 1348 interactions (Appendix S3 and S4). These two networks could be considered clusters of signaling proteins controling membrane protein tyrosine phosphorylation by Xk and chorein. Since both Xk and chorein lack intrinsic kinase or phosphatase activity, their influence on the tyrosine phosphoproteome must be mediated by interacting kinases and/or phosphatases. In the Xk_to_P-tyr-network we identified 29 kinases (Appendix S13); two kinases, ABL2 and MARK1 belonged to the set of hyperphosphorylated proteins (both +2.84); three kinases, CSNK2A1, PRKACB and PRKCA, were first neighbors of Xk and directly interacted with all other 24 kinases. We also identified three phosphatases (Appendix S13), DUSP13, SET and PTPRC; DUSP13 belonged to the set of hyperphosphorylated proteins (+2.17). Since we experimentally identified proteins phosphorylated on tyrosine residues, the relevant kinases were the PTKs ABL2 and FYN, whereas the relevant phosphatases were DUSP13 and PTPRC. As PTPRC expression is restricted to leukocytes, we focused our analysis on ABL2, FYN and DUSP13.

**Figure 1:**
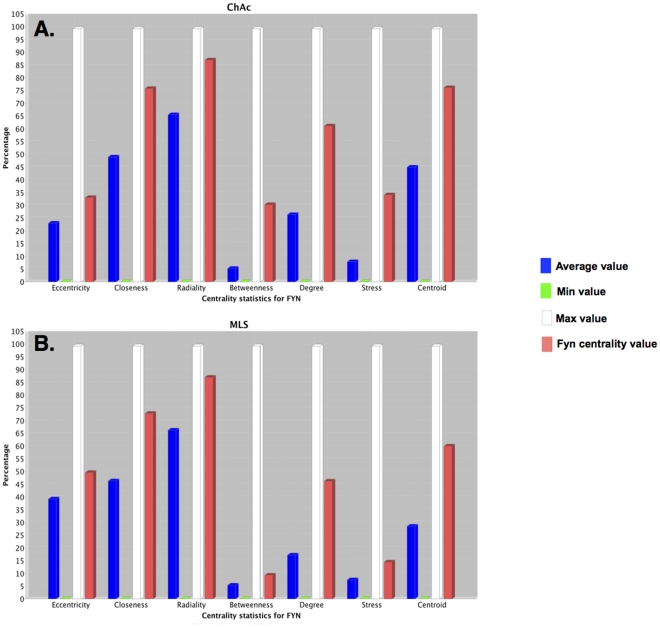
Scores of FYN centrality indexes in Xk_to_P-tyr- and chorein_to_P-tyr- networks. (**A**) Eccentricity, closeness, radiality, betweenness, degree, stress and centroid centrality indexes of the src-related, protein tyrosine kinase FYN in the chorea-acanthocytosis (ChAc; chorein_to_P-tyr)-related sub-network. (**B**) Eccentricity, closeness, radiality, betweenness, degree, stress and centroid centrality indexes of the src-related, protein tyrosine kinase FYN in McLeod syndrome (MLS; Xk_to_P-tyr)-related sub-network. The score of every index was normalized to the maximal value for every index, considered as 100%. Red columns are relative values for FYN. Blue columns are average values. White columns are maximal values. Green columns are minimal values.

**Figure 2:**
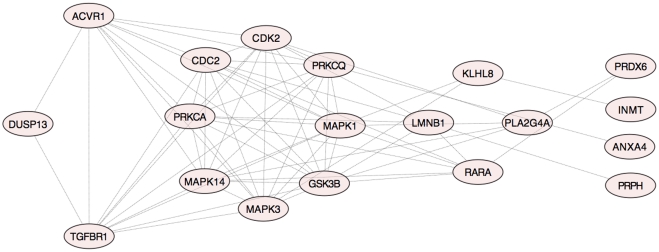
DUSP13 shortest paths to dephosphorylated proteins in Xk_to_P-tyr-network. Graph of all shortest paths linking DUSP13 to INMT, PRPH, PRDX6 and ANXA4. A distance of 3 (ANXA4) or 4 (INMT, PRPH, PRDX6) degree separates DUSP13 from the proteins found dephosphorylated in red cell from patients with McLeod syndrome (MLS).

To refine this first analysis we calculated the centrality scores for every node of the Xk_to_P-tyr-network (see Methods) (Appendix S14 and S15). This allowed us to rank nodes according to their topological weight in the network [12], [13], [14]. We found that all centrality indexes of FYN were well above the network average (Figure 1A). In particularly, the centroid was 2.1 times the average, betweenness was 1.7 times the average and stress was 1.92 times the average, thus ranking FYN in the top ten nodes. As the centroid indicates node tendency to organize functional modules, with betweenness and stress denoting the capability of a node to work as a critical linker between nodes, these elevated FYN centralities may suggest a critical regulatory role of FYN in this specific red cell context. In contrast, ABL2 had betweenness and stress well below the average, but centroid was over the average (Supplementary Figure S1). This may suggest a less critical role of ABL2 in maintaining node communication but still with a role in cluster formation. Finally, all centrality indexes of DUSP13 were well below the average (Supplementary Figure S2), suggesting a rather marginal role in network regulation. Calculation of shortest paths linking DUSP13 to the set of dephosphorylated proteins showed a rather indirect connection, with 3- to 4-degrees of separation (Figure 2, Appendix S5). Overall, the analysis suggests that FYN and ABL2 have a dominant topological role with respect to DUSP13. This suggests a dominant role in regulation of tyrosine phosphorylation in red cells from MLS patients in agreement with the observed hyperphosphorylating state of red cell membrane proteins in MLS. The previous conclusions are in accordance with previously reported data on other DUSP-related phosphatases, showing that phosphorylation of DUSPs may lead to their inhibition and/or degradation [15]. Four proteins were found to be dephosphorylated in red cells from MLS patients (ANXA4, PRPH, PRDX6 and INMT). Since the analysis suggests a marginal role of DUSP13, the only protein tyrosine phosphatase found by the analysis and possibly affecting protein tyrosine phosphorylation is PTPRC, which binds and modifies FYN activity [16]. Interestingly, at present no data are available on PTPRC expression in mature red cells.

ABL2 is another protein that can directly bind FYN and might be regulated by FYN-mediated phosphorylation of its SH2 and SH3 domains, suggesting a more complex scenario. In addition, FYN may interact with PRKCA [17], which may in turn directly interact with Xk. Finally, PTPRC binding CSNK2A1 and PRKCA may possibly participate in a multiprotein complex organized at the membrane by Xk. Altogether, these observations may indicate that a deficient Xk on red cells from MLS patients may alter the intracellular distribution and/or reciprocal interaction of FYN, ABL2, DUSP13 and, possibly, PTPRC. This promotes an imbalanced between kinases and phosphatases activity, leading in favour of FYN and ABL2.

In the chorein_to_P-tyr-network we identified 29 kinases (Appendix S16). Six kinases, AURKB (+2.6), CAMK1 (+2.5), PLK3 (+2.6), MPP1 (+2.6), PIP4K2A (+2.9) and PFTK1 (+2.4) belonged to the set of hyperphosphorylated proteins. Three kinases, FYN, ABL1 and PIK3R1, were first neighbors of chorein and directly interacted with all other 26 kinases. We also identified four phosphatases (Appendix S16), PPP3CB, PPP3CC, ACP1 and PTPRC; PPP3CC belonged to the set of hyperphosphorylated proteins (+2.2). Also in ChAc red cells we experimentally identified proteins phosphorylated on tyrosine residues. The relevant PTKs were FYN, ABL1, EGFR, FGFR1, IGF1R, TEC, TGFBR1 and BTK, whereas the relevant phosphatases were PTPRC and ACP1. As for the XK_to_P-tyr-network, we refined the analysis by calculating the centrality scores for every node of the chorein _to_P-tyr-network (Appendix S17 and S18), thus ranking nodes according to their topological relevance in the network. Among PTKs, FYN, ABL1 and EGFR had centralities values consistently above the average and ranked among the top ten nodes: (i) FYN had centroid 2.4 times the average, betweenness 5.6 times the average and stress 4.2 times the average; (ii) ABL1 had centroid 2.4 times the average, betweenness 6.6 times the average and stress 5.6 times the average (Supplementary Figure S3); (iii) EGFR had centroid 4 times the average, betweenness 5.5 times the average and stress 5 times the average (Supplementary Figure S4). In contrast, FGFR1, IGF1R, TEC, TGFBR1 and BTK had lower centrality scores and well below the network average (Supplementary Figure S5). Moreover, both identified phosphatases, PTPRC and ACP1 had very low centrality scores, suggesting a rather marginal role in network regulation with low influence on the tyrosine phosphoproteome (Supplementary Figure S5). Thus, centrality analysis not only suggests the absolute topological prevalence of FYN, ABL1 and EGFR over other PTKs but also on phosphatases in general. This is in agreement with the experimentally determined definitive tendency to protein hyperphosphorylation in ChAc red cells, with limited dephosphorylated proteins. During the analysis GRB2 attracted our attention. Indeed, GRB2 had the highest scores for all calculated centrality indexes in the chorein _to_P-tyr-network (Supplementary Figure S6). GRB2 is a direct interactor of chorein and directly interacts with FYN, ABL1, EGFR and PTPRC, and, through FYN and SPTAN1, with ACP1. Since FYN and ABL1 also directly interact with chorein, in ChAc red cells the absence of VPS13A may potentially affect the organization and localization of a signaling complex including GRB2, FYN, ABL1, EGFR, PTPRC, and ACP1. Due to the docking role of GRB2 (more than 700 signaling proteins, including several PTKs, directly dock on GRB2), it is possible that delocalization of GRB2 from chorein in red cells isolated from ChAc patients may modify the docking role of GRB2. This would lead to the generation of PTKs signaling complexes whose activity is not balanced by phosphatases. This conclusion seems supported by the absolutely prevalent topological relevance of FYN, ABL1 and EGFR versus PTPRC and ACP1 and by the interaction-phosphorylation relationships. Indeed, FYN directly interacts with ACTB (+2.3) and AURKB (+2.6); ABL1 directly interacts with ACTB (+2.3), AURKB (+2.6) and CAT (+2.5); ACP1 directly interact with EPB41 (+4.3).

Overall, this first analysis suggests that an altered plasma membrane localization of FYN, ABL1, ABL2 and EGFR, not balanced by a concurrent co-localization of ACP1 and PTPRC, may explain the pattern of protein tyrosine phosphorylation observed in red cells from MLS and ChAc patients.

### Combined network analysis and generation of shared regulatory cluster of kinases

The second step of our analysis was intended to unveil common signaling mechanisms shared by MLS and ChAc in acanthocytes. To this end, we first reconstructed two Xk- and chorein –“enriched” networks consisting of the previous Xk_to_P-tyr-network and chorein _to_P-tyr-network respectively combined with two networks generated by computing the first neighbors of the proteins belonging to the MLS and ChAc tyrosine phosphoproteomic data sets.

The Xk-PY_probe-FN network consisted of 316 nodes and 16702 interactions (Appendix S6); the chorein-PY_probe-FN network consisted of 930 nodes and 30351 interactions (Appendix S7). Upon network union, the Xk-enriched network consisted of 373 nodes and 17080 interactions (Appendix S8), whereas the chorein-enriched network consisted of 941 nodes and 30549 interactions (Appendix S9). These two networks represent interactomic spaces related to Xk and chorein signaling activities. To identify a common interactomic background shared by MLS and ChAc, we applied an intersection algorithm generating a unique, fully connected, interactomic network component. This Xk_ chorein-intersected network consisted of 249 nodes and 16131 interactions (Appendix S10). Every protein and interaction belonging to the Xk_ chorein-intersected network is present in both in Xk- and in chorein-union networks. This intersected network is likely the common interactomic space where shared signaling mechanisms may emerge.

To further focus the analysis we calculated the centrality score for every node of the Xk_ chorein-intersected network (Appendix S19 and S20, Appendix Figure 1). We, then, computed the most represented Gene Onthologies (GO) categories (www.geneontology.org) in the Xk_chorein-intersected network (Appendix S21). Finally, we filtered the Xk_ chorein-intersected network toward the node centralities scores, the GO categories and the biochemical activity. By applying this network filtering procedure we better focused the analysis and generated a number of interesting output. First, network filtering by kinase and phosphatases activity revealed 144 protein kinases but only 3 protein phosphatases, suggesting an absolutely imbalanced activity toward protein phosphorylation present in the Xk_chorein-intersected network. Notably, we found 4 proteins (ACTB, CAT, AURKB and RAB3C) hyperphosphorylated in ChAc red cells and 3 proteins (MARK1, ABL2, RPH3AL) hyperphosphorylated in MLS red cells.

**Figure 3:**
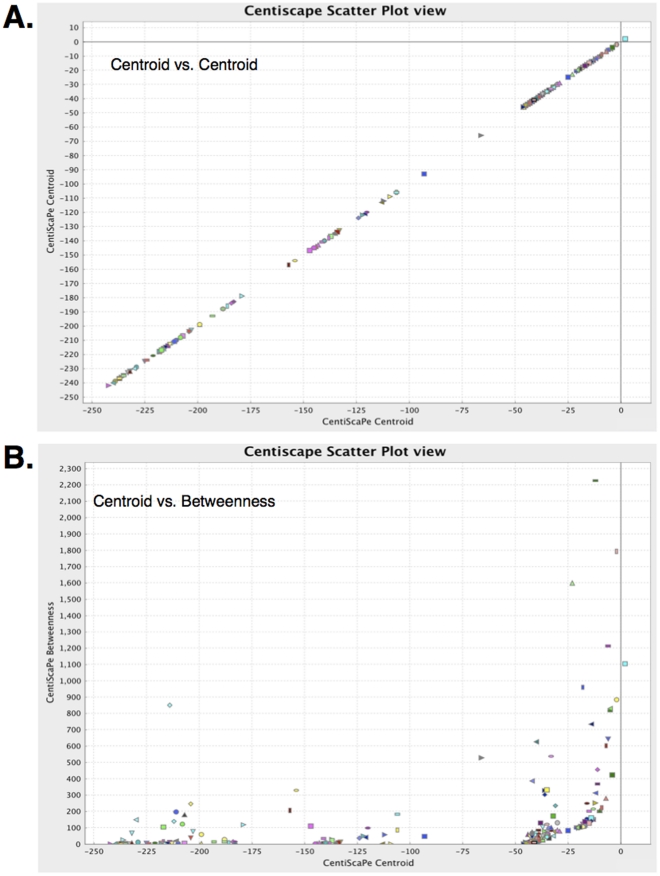
Centrality discretization in Xk_VPS13A-intersected network. (**A**) Plot of centroid vs. centroid centrality index values of all nodes in the Xk_ chorein-intersected network. (**B**) Plot of centroid vs. betweenness centrality index values of all nodes in the Xk_chorein-intersected network; the plot shows a concurrent discretization of centroid and betweenness, highlighting a cluster of 41 proteins having centroid and betweenness above the network average.

**Figure 4:**
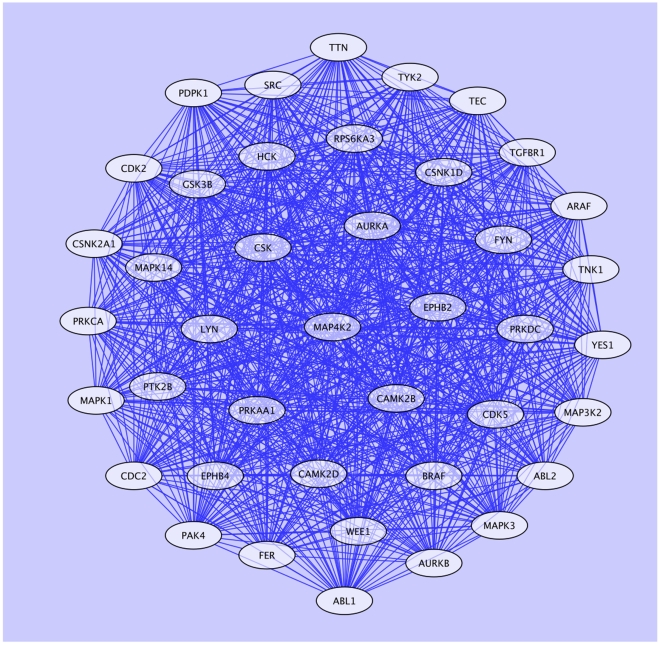
Sub-network of proteins in the Xk_ chorein-intersected network. Sub-network of proteins in the Xk_chorein-intersected network having centroid and betweenness over the network average. The sub-network contains 41 proteins connected by 818 interactions. All proteins are kinases.

**Figure 5:**
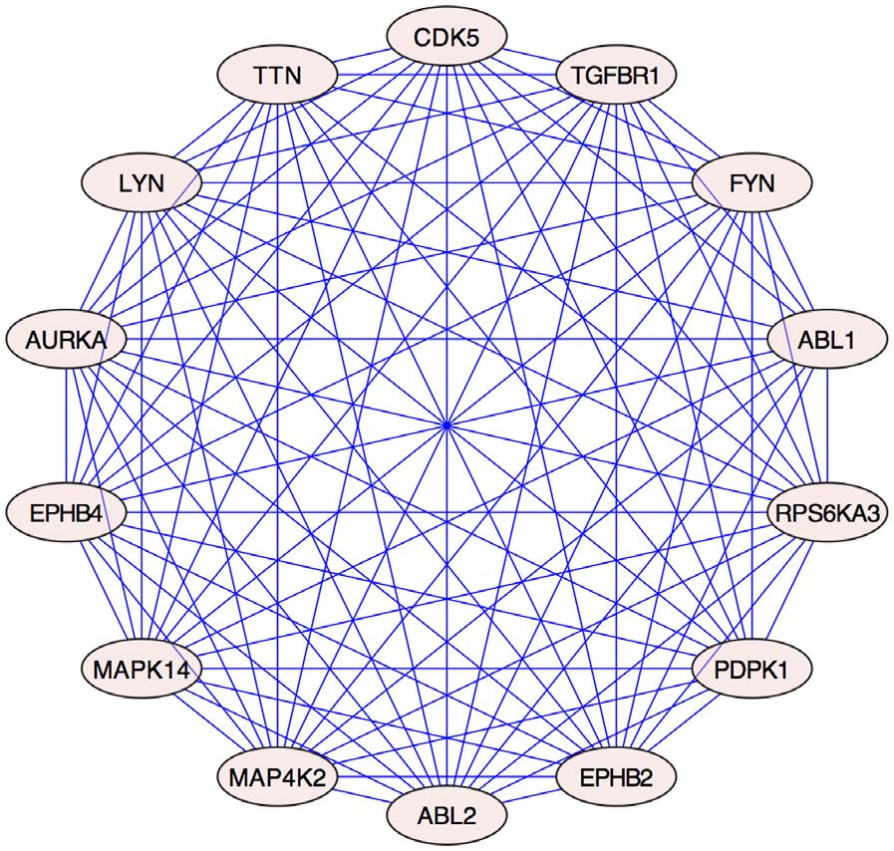
Highly restricted sub-network of, GO categorized, clustered proteins isolated from the Xk_chorein-intersected network. Sub-network of proteins in the Xk_chorein-intersected network having centroid and betweenness above the network average and enriched in the GO categories “erythrocyte development” and “neurogenesis”. The sub-network consists of 14 proteins connected by 89 interactions. All proteins are kinases.

Secondly, analysis of centroid demonstrated an organization of the Xk_chorein-intersected network in at least 4 main protein clusters (Figure 3A). This suggested the possibility of a further focusing of the analysis. We plotted centroid versus betweenness (Figure 3B) to evidence a concurrent discretization of the betweenness. We then filtered out the Xk_chorein-intersected network by extracting a sub-network of nodes having both centroid and betweenness above the average (far and top right of the Figure 3B; Appendix S11). All proteins belonging to this sub-network (41 nodes - 818 interactions) were kinases (Figure 4, Appendix S22). We finally extracted all proteins from this sub-network, which show the highest statistical scores in the GO categories “erythrocyte development” and “neurogenesis”. The resulting highly restricted sub-network consisted of 14 proteins and 89 interactions (Figure 5, Appendix S12, Appendix S23 and S24). This network was extremely connected with an average shortest path of 1, a neighborhood connectivity of 12 and a clustering coefficient of 1, indicating that this network works as a unique, fully integrated, functional signaling module. The network included ABL2, FYN and ABL1 along with other 11 kinases. FYN, LYN, ABL2, TTN and PDPK1 are involved in rho small GTPases activity and cytoskeleton regulation (see http://www.signaling-gateway.org/molecule and http://www.geneontology.org/); RPS6KA3, EPHB2, EPHB4 and CDK5 regulate neurogenesis (see http://www.signaling-gateway.org/molecule and http://www.geneontology.org/); TGFBR1 regulates development; MAP4K2 and MAPK14 are involved in response to stress (see http://www.signaling-gateway.org/molecule and http://www.geneontology.org/); LYN regulate erythrocyte differentiation and band 3 tyrosine phosphorylation state [18], [19]; ABL1 and ABL2 are related to oxidoreductase activity and oxidative stress. Of particular interest is the presence in this highly connected sub-network of TTN (Titin), which is a muscle giant (4.3 MD) scaffolding protein characterized by intrinsic viscous-elastic stiffness and kinase activity, involved in cytoskeleton regulation and contractility in the sarcomere [20].

Interrogation of the 8 manually curated data-bases from which we derived the interactomic data-set used in this study (HPRD, BioGRID, MINT, IntAct, Reactome, CELL-MAP, NCI_Nature and Pathway Commons), show interactions between the 14 proteins described in various publications. Interactions have been shown both *in vitro* (cell-free system) and/or *in vivo* depending of data-bases, some representative references are shown in Appendix S25.

Overall, the two combined analyses point to a very restricted group of highly interconnected kinases including ABL1, ABL2, AURKA, CDK5, EPHB2, EPHB4, FYN, LYN, MAP4K2, MAPK14, PDPK1, RPS6KA3, TGFBR1 and TTN (Figure 5, see Appendix S24), regulating rho small GTPase-mediated signaling, cytoskeleton network, erythropoiesis and neurogenesis. This network may represent a shared regulatory cluster of kinases whose alteration is most likely involved in generation of the abnormal red cells that characterize MLS and ChAc.

## Discussion

The maintenance of the red cell membrane mechanical stability is crucial for red cell functions and survival in peripheral circulation. The mechanisms involved in this process are complex and only partially understood. Recent proteomic studies have identified more than 300 erythrocyte membrane proteins indicating that most of the available information is limited to less than 15% of total membrane proteins [8], [21], [22], [23], [24]. In addition, changes in phosphorylation state of some of the most abundant red cell membrane proteins have been reported to affect the red cell membrane organization with loss of mechanical membrane stability and abnormal red cell morphology [7], [8], [9]. This indicates that the red cell membrane contains a consistent number of regulatory structures characterized by unexpected complexity. The events involved in generation of acanthocytes associated with NA syndromes are only partially known. In this context, a proteomic approach enable the rapid identification of new functional pathways. In previous reports we have shown abnormalities in protein tyrosine-phosphorylation state of few red cell membrane proteins, suggesting abnormalities of intracellular signaling pathways involved in acanthocytosis in NA [11], [25].

To infer potential mechanisms of disease shared by MLS and ChAc we performed a bioinformatic analysis of the experimentally-determined Tyr-phosphoproteomic data sets by implementing an articulated procedure of topological network analysis [26], [27], [28], [29]. In the first level of analysis when the phosphoproteomic data from MLS and ChAc were separately reconstructed and analyzed, we observed an imbalance between kinase/phosphatase membrane translocation compared to controls. The centrality index analysis suggests that the absolute topological prevalence of few tyrosine kinases, such as FYN and ABL1, over other PTKs may contribute to the imbalanced activity between kinases and phosphatases in acanthocytes. This is in agreement with the experimentally determined definitive tendency to protein hyperphosphorylation in ChAc red cells, with limited dephosphorylated proteins. Notably, the analysis suggested a negligible topological role for ACP1, a low molecular weight protein tyrosine phosphatase, in the ChAc signaling network. In other cell models ACP1 may modulate the activation state of rho small GTPases [30], which in turn are involved in cytoskeleton remodeling [31], [32]. The recent data on erythrocytes from mice genetically lacking the small G protein Rac1 indicate that these proteins are involved in the dynamic regulation of the red cell membrane network [33]. This suggests that a perturbation in the events involved in cytoskeleton rearrangement might participate to the generation of acanthocytes in ChAc and MLS. Based on this analysis we obtained preliminary data showing increased membrane association of small G proteins in ChAc red cells compared to normal controls, supporting the network modeling [34].

The topological network analysis of red cells from MLS patients indicated that a defective Xk protein on red cells may possibly alter the intracellular distribution and/or reciprocal interaction of FYN, ABL2, DUSP13 and, possibly, PTPRC. This would generate an imbalanced activity of tyrosine kinases versus phosphatases in MLS also. To address whether shared signaling mechanisms operate in acanthocytes from MLS and ChAc, NA disorders caused by different genes, we finally combined the two network analyses. We found a very restricted group of highly interconnected kinases including ABL1, ABL2, AURKA, CDK5, EPHB2, EPHB4, FYN, LYN, MAP4K2, MAPK14, PDPK1, RPS6KA3, TGFBR1 and TTN (Figure 5, see Appendix Table 12), regulating rho small GTPases-mediated signaling, cytoskeleton network, erythropoiesis and neurogenesis. This network could likely represent a shared regulatory cluster of kinases whose alteration is responsible for abnormal red cells in MLS and ChAc.

Previous studies have shown that FYN and LYN, two tyrosine kinase of the Src family, are present in red cells [7], [19]. Alteration of Fyn activity and generation of red cells with abnormal morphology has been previously described in a mouse model genetically lacking the protein tyrosine phosphatase epsilon (PTPε).This supports the crucial role of FYN in modulating the tyrosine phosphorylation state of red cell membrane protein and emphasizes the balance between phosphatase and kinase activities in the maintenance of red cell membrane mechanical stability [7]. The identification of LYN as another candidate of this shared signaling network is very interesting because of the increased tyrosine phosphorylation state of band 3 reported in red cells from ChAc [7], [25]. In normal red cells, the translocation of functionally active LYN to red cell membrane is a sequential process of phosphorylation synergistically mediated by SYK, a ZAP-70 related tyrosine kinase. SYK generates a binding site for the Src SH2 domain followed by LYN membrane association [19]. It is of interest that SYK is not present in the MLS and ChAc-related shared kinase network, suggesting either abnormal LYN activation, possibly SYK-independent as we recently reported in ChAc red cells [34], or changes in the accessibility of the membrane docking site for LYN in abnormal red cells from ChAc and MLS patients.

Overall, we believe that the results of the present work may impact the study of signaling networks in red cells. Indeed, dynamics of protein-protein kinase interactions could provide the molecular context necessary for cell plasticity, in turn required to maintain erythrocyte shape, steadiness and for long-term survival of red cells. This aspect needs to be investigated especially in diseased red cells. Our results provide a proof-of-principle for a potentially useful bioinformatic approach by combining several algorithms of topological network analysis coupled to multidimensional network node categorization to allow consistent filtering of network complexity. We believe that this approach may accelerate the progress in kinome characterization in MLS and ChAc diseases and will possibly become informative for the analysis of signaling networks in normal and diseased red cells.

## Materials and Methods

### Proteomic data generation

We studied nine patients with chorea-acanthocytosis (ChAc) and five patients with McLeod syndrome (MLS), in whom the diagnosis was based on the clinical neurological manifestation, the presence of acanthocytes and the molecular analysis of either *VSPA13* (chorein) or *XK* (Xk) mutations as previously described [1], [2], [3], [4], [5], [35]. Table 1 reports the demographic, clinical and molecular data of the patients studied [5], [11], [35], [36], [37], [38], [39], [40], [41], [42], [43]. The study was approved by either Ethic Committees or the Istitutional review boards for human subject study of the James J. Peters VAMC (Bronx, NY, USA), of Mount Sinai School of Medicine (NYC, NY, USA), of Zurich University (Zurich, Switzerland) and of Siena University (Siena, Italy). Blood was collected after informed written consent had been obtained from each subject. Plasma and buffy coat were removed after centrifugation at 1,200 *g* for 10 minutes and the cells were washed four times with a choline washing solution containing: 152 mmol/L choline chloride, 1 mmol/L MgCl2, 10 mmol/L TRIS-MOPS, pH 7.40 at 4°C as previously described [44]. Packed red cells were lysed in ice cold Phosphate Lysis Buffer (LB: 5 mM Na2HPO4, pH 8 containing: protease inhibitor cocktail tablets (Roche), 3 mM benzamidine, final concentration) and centrifuged 10 min at 4°C at 12,000 *g*. The red cell membrane were washed several times in LB and protein content was quantified using DC Protein Assay (Biorad) [7]. The red cell membrane proteins were separated by bidimensional electrophoresis as previously reported [7], [8] and the 2D colloidal Coommassie stained gels were scanned and digitized with Progenesis Same Spots software (Nonlinear Dynamics, Newcastle-Upon-Tyne, UK) to generate a general pattern in the 3–10 pH rage. Using the twofold abundance change criteria combined with one-way ANOVA analysis we found 91 spots differently expressed in ChAc *vs* control as previously reported [34] and 28 spots differently expressed in MLS *vs* control (Supplementary Table S1). The spots differently expressed were identified by MALDI-TOF MS analysis as previously described [7], [8]. The tyrosine phosphorylation profile of ChAc and MLS red cell membrane was analyzed as previously reported [7], [8]. The bands or spots differently tyrosine phosphorylated were identified by Progenesis Same Spots software (Nonlinear Dynamics, Newcastle-Upon-Tyne, UK) following the densitometric analysis of the scanned images of unsaturated films (ImageJ v 1.28 software). The selected bands or spots were identified by MALDI-TOF MS/MS [7], [8] are shown in Supplementary Table S2, S3.

### Building of a Global Mammalian Protein Interactomic network

A Global Mammalian Protein Interactomic network was built by combining the interactomic data sets from HPRD, MINT, BioGrid, IntAct, DIP, BIND and Pathway Commons online databases, complemented by in-house manually curated data derived from the literature. The combined data set included only manually curated protein-protein binary interactions, inferred by two to six independent methods. Functional, protein-DNA, protein-RNA, protein-metabolite and protein-drug interactions, eventually present in the data sets, were removed. To avoid miscalculations of topological parameters, duplicates and self-interactions, leading to self-loops, were also removed. All molecule identifiers were normalized to HGNC official symbols (www.genenames.org) by using Babelomics 4 (http://babelomics.bioinfo.cipf.es/index.html) and Clone/Gene ID converter (http://idconverter.bioinfo.cnio.es/). The entire set of gene IDs derived from HGNC was also incorporated in the interactomic set to facilitate full interrogation of the data set, thus considering proteins for which direct, physical, interactions were still not described. The resulting data set consisted of 29012 unique gene IDs (nodes) and 149967 binary interactions (edges). The data set contained a PPI network consisting of a unique connected giant component including 11976 nodes (all proteins) and 149879 edges, 68 isolated sub-components, including a total of 159 nodes and 88 edges connected by a maximum of 5 to a minimum of 2 edges, and 16877 isolated nodes. 20451 edges were functionally tagged, with 18286 directed edges accounting for the biochemical activity “state change”, and 2165 undirected edges accounting for the biochemical activity “complex formation”. Where unambiguously defined, the category “state change” was further specified as “activation” or “inhibition”. 94% of total interactions were human and 6% were mouse and rat interactions. The combined data set was compiled in .sif (network) and .txt (attribute) formats to be analyzed in the network analysis environment Cytoscape (www.cytoscape.org) [45], [46]. This interactomic data set can be considered a sort of virtual cell including all known protein-protein binary interactions and represents the protein interactomic context where cell regulation originates.

### Node annotation and mapping

Protein annotations were extracted from protein annotation data files obtained from HPRD, BioGRID, HGNC and GO databases. Annotations were mapped to all proteins present in the Global Mammalian Protein Interactomic network upon normalization to HGNC protein IDs and were compiled in a single TAB-delimited file to be loaded, as a multidimensional layer of protein properties, in the network analysis software Cytoscape. Node annotations were used to filter sub-networks versus specific protein biochemical or functional properties thus allowing further functional focusing of the analysis.

### Sub-network reconstruction and analysis

To perform network topological analysis in the context of MLS and ChAc, the two experimentally determined sets of phosphorylated proteins, derived form the analysis of Red cells isolated from MLS and ChAc patients, were considered as “bioinformatic probes”. These were used to interrogate the Global Mammalian Protein Interactomic network and extract enriched sub-networks of proteins related to the activity of Xk or chorein proteins and of the two tyr-phosphoproteomic sets. To perform the reconstruction of MLS and ChAc. PPI sub-networks, we used standard tools of network interrogation, reconstruction, filtering and manipulation provided by the core code of the Cytoscape network analysis environment. Additionally, we applied computational features provided by dedicated plug-ins.

To calculate all shortest paths linking Xk and chorein to the corresponding set of phosphorylated proteins, we developed the Cytoscape plug-in Pesca 3.0 (under submission). The plug-in computes all the shortest paths from a root node to all the other nodes in the network. Formally, given a network described as an undirected graph *G = (N,E)*, where *N* is the set of nodes and *E* is the set of edges, a path from node *n_1_* to node *n_k_* is an alternative sequence *n_0_,e_1_,n_1_,e_2_,n_2_,…,e_k_,n_k_* such that *e_i_ = {n_i_,n_i_+1}*, where *e_i_≠e_j_*, if *i≠j.* The length of a path is the number of edges it has. A path *P* from a source node *s* to a target node *t* is the shortest path if its length is the smallest possible among all paths from *s* to *t*. The algorithm used by the Pesca 3.0 plugin to find the shortest paths is based on the Dijkstra algorithm [47]. To compute all the shortest paths the Dijkstra algorithm has been adjusted as follows. Exploring the graph when calculating the shortest path between two nodes *s* and *t*, the Dijkstra algorithm keeps for each node *n* a predecessor node *p*. To have all the shortest paths, we replace the predecessor *p* with a set of predecessors for each node *n*. The set of predecessors of the node *n* is the set of all the predecessors of the node *n* in the shortest paths set between *s* and *t*, i.e. one node is in the set of predecessors of *n* if and only if it is a predecessor of *n* in one of the shortest paths between *s* and *t* containing *n*. Once the predecessors set of each node *n* has been computed, the tree of all the shortest paths between *s* and *t* can also be easily computed.

In biological term, the shortest path is the minimal path of functional influence of an agent A on a target B. Thus, the shorter the path the more likely is the functional influence of A on B. This principle was applied to generate the two MLS- and ChAC-related clusters of signaling proteins most likely mediating protein phosphorylation by Xk and chorein. To gain maximal significance, the network reconstruction based on direct interactor identification was limited to the first neighbor determination. Thus, only first neighbors of the proteins belonging to the two phosphoproteomic data sets were extracted from the Global Mammalian Protein Interactomic data set. This procedure generated the most likely cluster of signaling proteins functionally related to the activity of the proteins belonging to the phosphoproteomic data sets.

Protein categorization based on topological relevance in the network was performed by means of centrality index calculation. The analysis of network centrality indexes was performed by computing node-by-node centrality scores with the Cytoscape plug-in CentiScaPe [12]. Node centrality indexes are complex topological parameters allowing quantitative local measurement of the position of a node relative to the other nodes, and can be used to infer node relative importance in global network organization. Thus, centrality index calculation allows categorization of nodes in a network according to their specific regulatory relevance with respect to other nodes in a network. Particularly we focused on betweenness, stress and centroid indexes (Table 2). Scatter plots of centrality scores were generated within CentiScaPe. Determination of Gene Ontology (GO) categories and calculation of statistical prevalence within the extracted sub-networks were automatically generated by applying the two Cytoscape plug-ins BiNGO [48] and ClueGO [49].

**Table 2:** Stress, Betweenness and Centroid centrality indexes.

Centralities definitionsσ*_st_*_ is the number of shortest paths from node *s* to node *t*_σ*_st_*_ (*v*) is the number of shortest paths from node *s* to node *t* passing through node *v*_	
Stress(*v*)	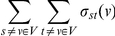
Betweenness(*v*)	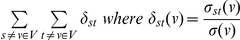
Centroid(*v*)	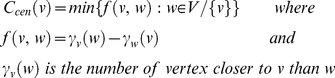

## Supporting Information

Figure S1 **Scores of ABL2 centrality indexes in the XK_to_P-tyr-network.** Eccentricity, closeness, radiality, betweenness, degree, stress and centroid centrality indexes of the protein tyrosine kinase ABL2 in the McLeod syndrome (XK_to_P-tyr)-related sub-network. The score of every index was normalized to the maximal value for every index, considered as 100%. Red columns are relative values for ABL2. Blue columns are average values. White columns are maximal values. Green columns are minimal values.
(TIFF)

Figure S2 **Scores of DUSP13 centrality indexes in the XK_to_P-tyr-network.** Eccentricity, closeness, radiality, betweenness, degree, stress and centroid centrality indexes of the protein tyrosine phosphatase DUSP13 in the Mcleod syndrome (XK_to_P-tyr)-related sub-network. The score of every index was normalized to the maximal value for every index, considered as 100%. Red columns are relative values for DUSP13. Blue columns are average values. White columns are maximal values. Green columns are minimal values.
(TIFF)

Figure S3 **Scores of ABL1 centrality indexes in the VPS13A_to_P-tyr-network.** Eccentricity, closeness, radiality, betweenness, degree, stress and centroid centrality indexes of the protein tyrosine kinase ABL1 in the ChAc (VPS13A_to_P-tyr)-related sub-network. The score of every index was normalized to the maximal value for every index, considered as 100%. Red columns are relative values for ABL1. Blue columns are average values. White columns are maximal values. Green columns are minimal values.
(TIFF)

Figure S4 **Scores of EGFR centrality indexes in the VPS13A_to_P-tyr-network.** Eccentricity, closeness, radiality, betweenness, degree, stress and centroid centrality indexes of the protein tyrosine kinase EGFR in the ChAc (VPS13A_to_P-tyr)-related sub-network. The score of every index was normalized to the maximal value for every index, considered as 100%. Red columns are relative values for EGFR. Blue columns are average values. White columns are maximal values. Green columns are minimal values.
(TIFF)

Figure S5 **Scores of FGFR1, IGF1R, TEC, TGFBR1, BTK, PTPRC and ACP1 centrality indexes in the VPS13A_to_P-tyr-network.** Eccentricity, closeness, radiality, betweenness, degree, stress and centroid centrality indexes of the protein tyrosine kinases FGFR1, IGF1R, TEC, TGFBR1, BTK, and protein tyrosine phosphatases PTPRC, ACP1 in the ChAc (VPS13A_to_P-tyr)-related sub-network. The score of every index was normalized to the maximal value for every index, considered as 100%. Red columns are relative values for FGFR1, IGF1R, TEC, TGFBR1, BTK, PTPRC and ACP1. Blue columns are average values. White columns are maximal values. Green columns are minimal values.
(TIFF)

Figure S6 **Scores of GRB2 centrality indexes in the VPS13A_to_P-tyr-network.** Eccentricity, closeness, radiality, betweenness, degree, stress and centroid centrality indexes of the docking protein GRB2 in the ChAc (VPS13A_to_P-tyr)-related sub-network. The score of every index was normalized to the maximal value for every index, considered as 100%. Red columns are relative values for GRB2. Blue columns are average values. White columns are maximal values. Green columns are minimal values.
(TIFF)

Table S1 List of identified proteins in comparative analysis between control and McLeod red cell membrane.
(DOC)

Table S2 List of identified proteins displaying different degrees of tyrosine phosphorylation in control and chorea-acanthocytosis red cell membrane.
(DOC)

Table S3 List of identified proteins displaying different degrees of tyrosine phosphorylation in control and McLeod red cell membrane.
(DOC)

Appendix S1 Network of proteins connecting, by means of shortest paths, Xk to proteins whose phosphorylation in tyrosine was found altered in RBCs from McLeod patients.
(TXT)

Appendix S2 List of all shortest paths connecting Xk to proteins whose phosphorylation in tyrosine was found altered in RBCs from McLeod patients.
(TXT)

Appendix S3 Network of proteins connecting, by means of shortest paths, chorein to proteins whose phosphorylation in tyrosine was found altered in RBCs from ChAc patients.
(TXT)

Appendix S4 List of all shortest paths connecting chorein to proteins whose phosphorylation in tyrosine was found altered in RBCs from ChAc patients.
(TXT)

Appendix S5 Network cluster of proteins linking DUSP13 to proteins found de-phosphorylated in tyrosine in RBCs from McLeod patients.
(TXT)

Appendix S6 Network of proteins including Xk and all proteins whose phosphorylation was found altered in RBCs from McLeod patients, expanded to the first neighbor (FN).
(TXT)

Appendix S7 Network of proteins including chorein and all proteins whose phosphorylation was found altered in RBCs from ChAc patients, expanded to the first neighbor (FN).
(TXT)

Appendix S8 Network derived form fusion of Appendix S1 and Appendix S6 McLeod networks.
(TXT)

Appendix S9 Network derived form fusion of Appendix S3 and Appendix S7 ChAc networks.
(TXT)

Appendix S10 Connected network derived from the intersection between Appendix S8 and Appendix S9 networks. This network only includes proteins relevant to both McLeod and ChAc RBCs phenotypes.
(TXT)

Appendix S11 Network as in Appendix S10, but reduced to include only proteins having both centroid and betweenness centrality indexes over the total network average.
(TXT)

Appendix S12 Network as in Appendix S11, but reduced to include only proteins having gene ontology (GO) attributes in the domains: erythrocyte development; neurogenesis (shown in Figure 5).
(TXT)

Appendix S13 Table of protein attributes for Appendix S1 network in the categories: Approved Name, Name Aliases, Chromosome, Entrez Gene ID, Kinases, MLS_PY, Phosphatases.
(TXT)

Appendix S14 Table of protein attributes for Appendix S1 network in the category: Node centrality indexes.
(TXT)

Appendix S15 Table of attributes for Appendix S1 network in the category: Network centrality indexes.
(TXT)

Appendix S16 Table of protein attributes for Appendix S3 network in the categories: Approved Name, Name Aliases, Chromosome, Entrez Gene ID, Kinases, MLS_PY, Phosphatases.
(TXT)

Appendix S17 Table of protein attributes for Appendix S3 network in the category: Node centrality indexes.
(TXT)

Appendix S18 Table of attributes for Appendix S3 network in the category: Network centrality indexes.
(TXT)

Appendix S19 Table of protein attributes for Appendix S10 network in the category: Node centrality indexes.
(TXT)

Appendix S20 Table of attributes for Appendix S10 network in the category: Network centrality indexes.
(TXT)

Appendix S21 Table of protein attributes for Appendix S10 network in the categories: GO BIOLOGICAL_PROCESS.
(TXT)

Appendix S22 Table of protein attributes for Appendix S10 network in the categories: Approved Name, Kinases, Phosphatases, ChAC_PY, MLS_PY, Bottom_acanthocytes, Betweenness, Centroid, Closeness, Eccentricity, Node degree, Radiality, Stress.
(TXT)

Appendix S23 Table of protein attributes for Appendix S11 network in the categories: Approved Name, GO BIOLOGICAL_PROCESS.
(TXT)

Appendix S24 Table of protein attributes for Appendix S12 network (shown in Figure 5) in the categories: Approved Name, Kinases, Phosphatases, ChAC_PY, MLS_PY, Bottom_acanthocytes, Betweenness, Centroid, Closeness, Eccentricity, Node degree, Radiality, Stress.
(TXT)

Appendix S25 Table of protein attributes for Appendix S12 network (shown in Figure 5) in the categories: in vitro evidence of interaction, in vivo evidence of interaction, PubMed reference.
(TXT)
