# Supplementary figures and images for: Computational Identification of Phospho-Tyrosine Sub-Networks Related to Acanthocyte Generation in Neuroacanthocytosis
Source: PLoS One. 2012 Feb 15;7(2):e31015. doi: 10.1371/journal.pone.0031015 (PMC3280254; doi:10.1371/journal.pone.0031015)

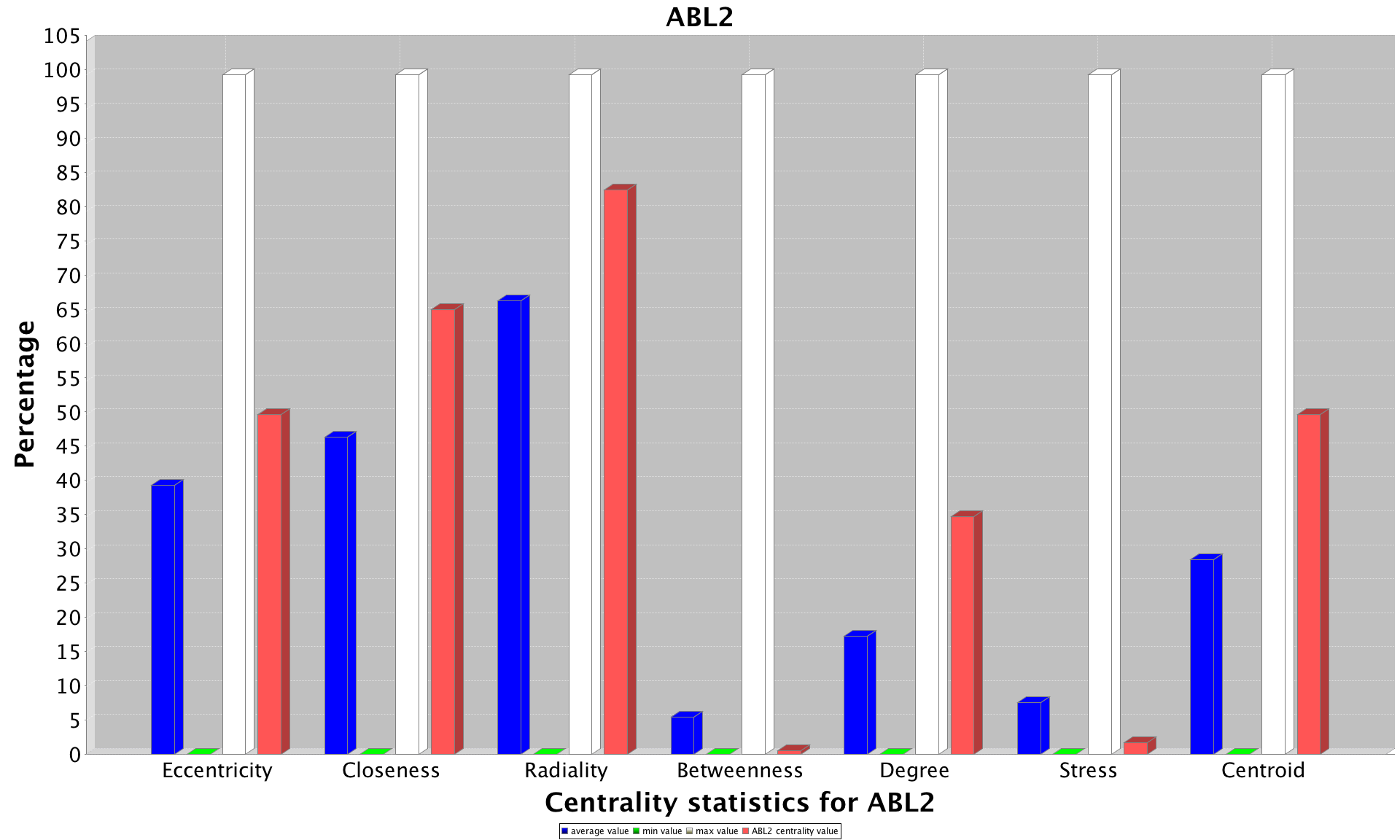

Supplement: Figure S1 — Scores of ABL2 centrality indexes in the XK_to_P-tyr-network. Eccentricity, closeness, radiality, betweenness, degree, stress and centroid centrality indexes of the protein tyrosine kinase ABL2 in the McLeod syndrome (XK_to_P-tyr)-related sub-network. The score of every index was normalized to the maximal value for every index, considered as 100%. Red columns are relative values for ABL2. Blue columns are average values. White columns are maximal values. Green columns are minimal values. (TIFF) [file pone.0031015.s001.tiff]

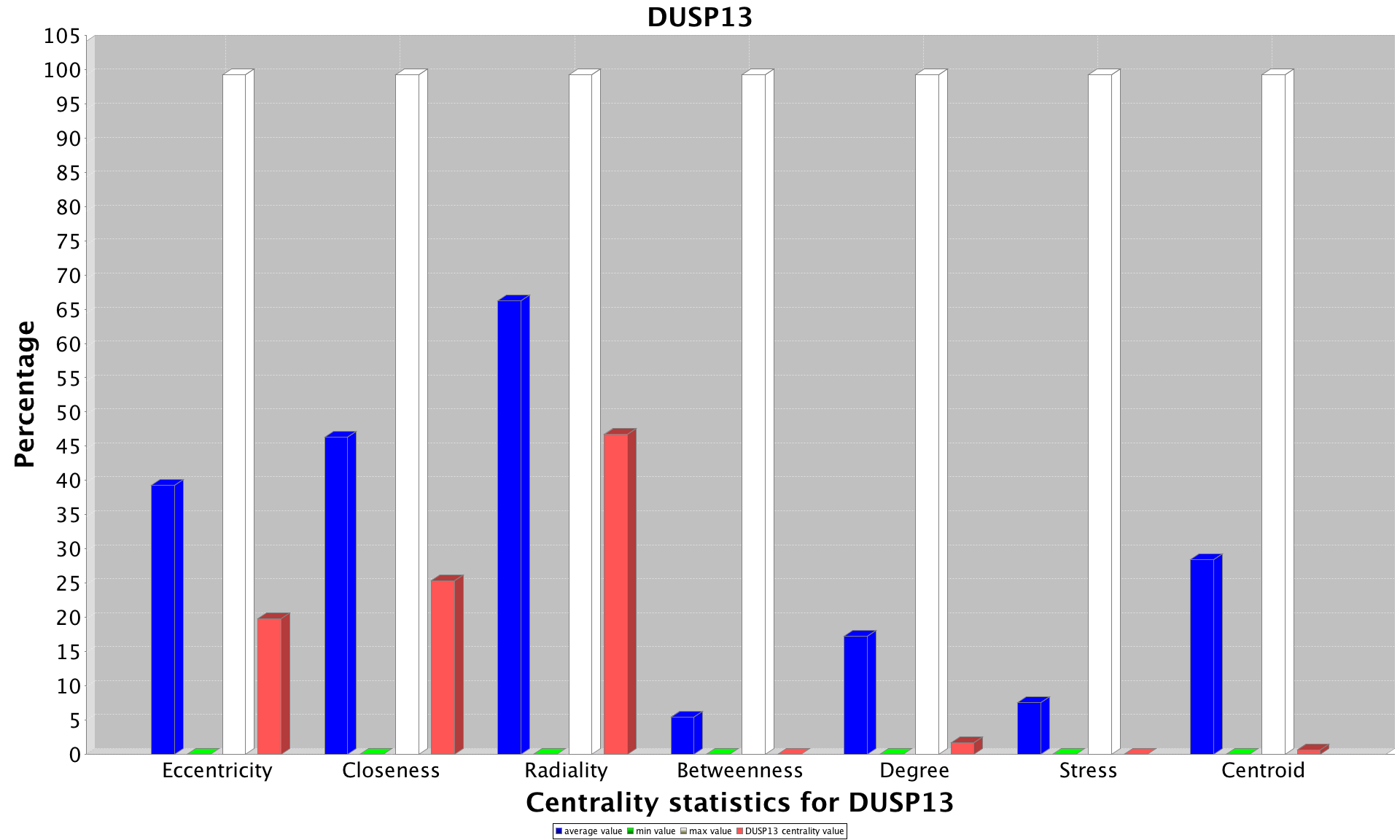

Supplement: Figure S2 — Scores of DUSP13 centrality indexes in the XK_to_P-tyr-network. Eccentricity, closeness, radiality, betweenness, degree, stress and centroid centrality indexes of the protein tyrosine phosphatase DUSP13 in the Mcleod syndrome (XK_to_P-tyr)-related sub-network. The score of every index was normalized to the maximal value for every index, considered as 100%. Red columns are relative values for DUSP13. Blue columns are average values. White columns are maximal values. Green columns are minimal values. (TIFF) [file pone.0031015.s002.tiff]

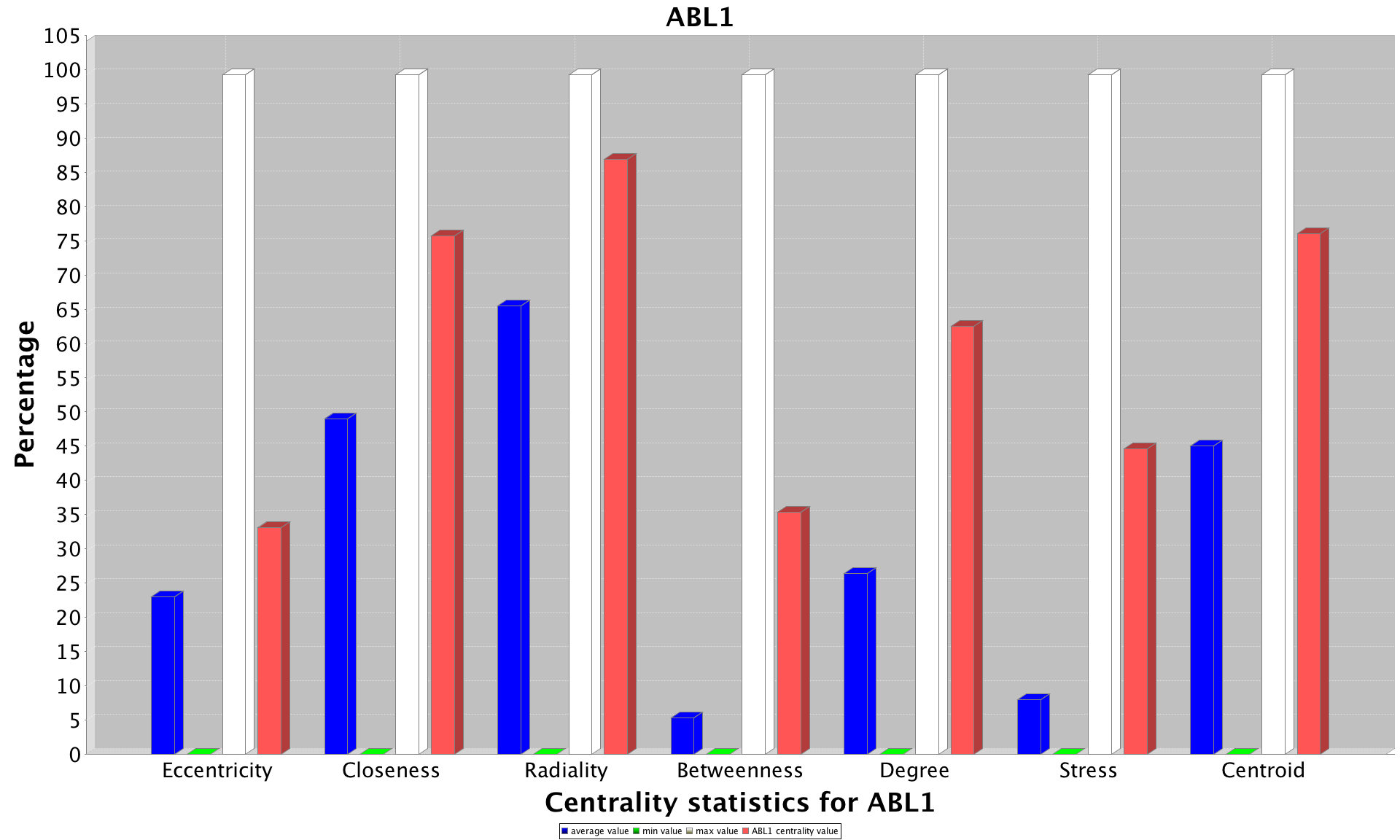

Supplement: Figure S3 — Scores of ABL1 centrality indexes in the VPS13A_to_P-tyr-network. Eccentricity, closeness, radiality, betweenness, degree, stress and centroid centrality indexes of the protein tyrosine kinase ABL1 in the ChAc (VPS13A_to_P-tyr)-related sub-network. The score of every index was normalized to the maximal value for every index, considered as 100%. Red columns are relative values for ABL1. Blue columns are average values. White columns are maximal values. Green columns are minimal values. (TIFF) [file pone.0031015.s003.tiff]

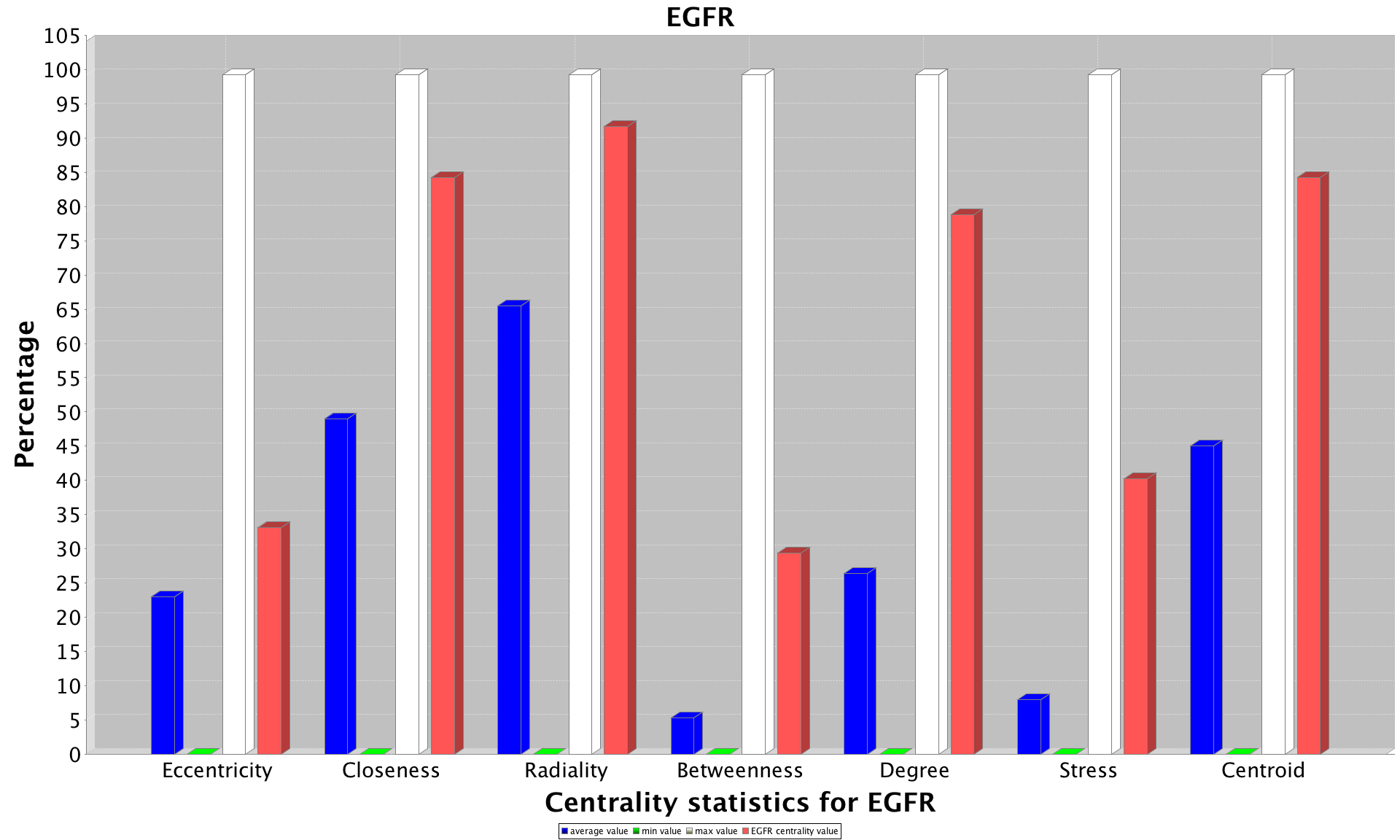

Supplement: Figure S4 — Scores of EGFR centrality indexes in the VPS13A_to_P-tyr-network. Eccentricity, closeness, radiality, betweenness, degree, stress and centroid centrality indexes of the protein tyrosine kinase EGFR in the ChAc (VPS13A_to_P-tyr)-related sub-network. The score of every index was normalized to the maximal value for every index, considered as 100%. Red columns are relative values for EGFR. Blue columns are average values. White columns are maximal values. Green columns are minimal values. (TIFF) [file pone.0031015.s004.tiff]

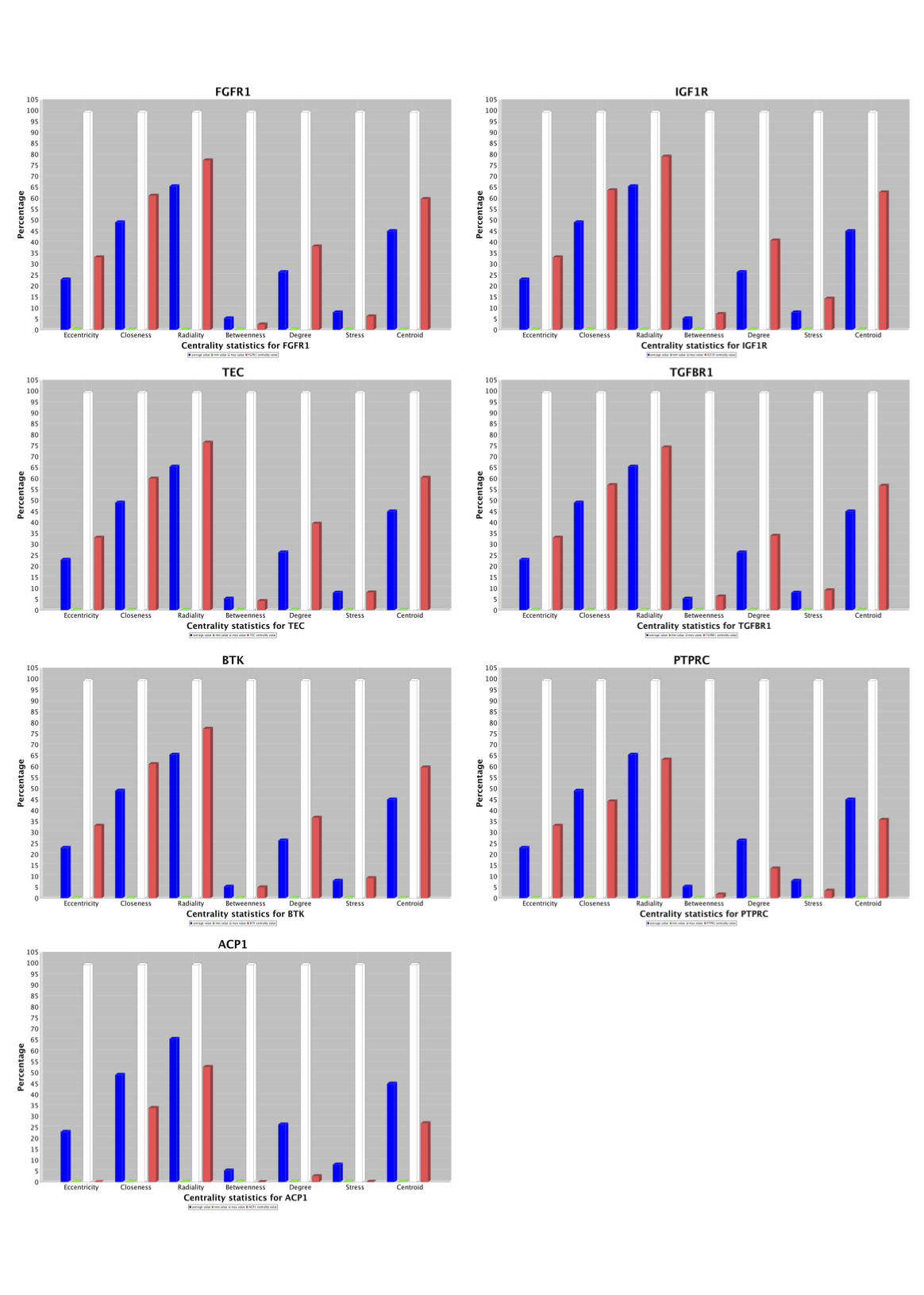

Supplement: Figure S5 — Scores of FGFR1, IGF1R, TEC, TGFBR1, BTK, PTPRC and ACP1 centrality indexes in the VPS13A_to_P-tyr-network. Eccentricity, closeness, radiality, betweenness, degree, stress and centroid centrality indexes of the protein tyrosine kinases FGFR1, IGF1R, TEC, TGFBR1, BTK, and protein tyrosine phosphatases PTPRC, ACP1 in the ChAc (VPS13A_to_P-tyr)-related sub-network. The score of every index was normalized to the maximal value for every index, considered as 100%. Red columns are relative values for FGFR1, IGF1R, TEC, TGFBR1, BTK, PTPRC and ACP1. Blue columns are average values. White columns are maximal values. Green columns are minimal values. (TIFF) [file pone.0031015.s005.tiff]

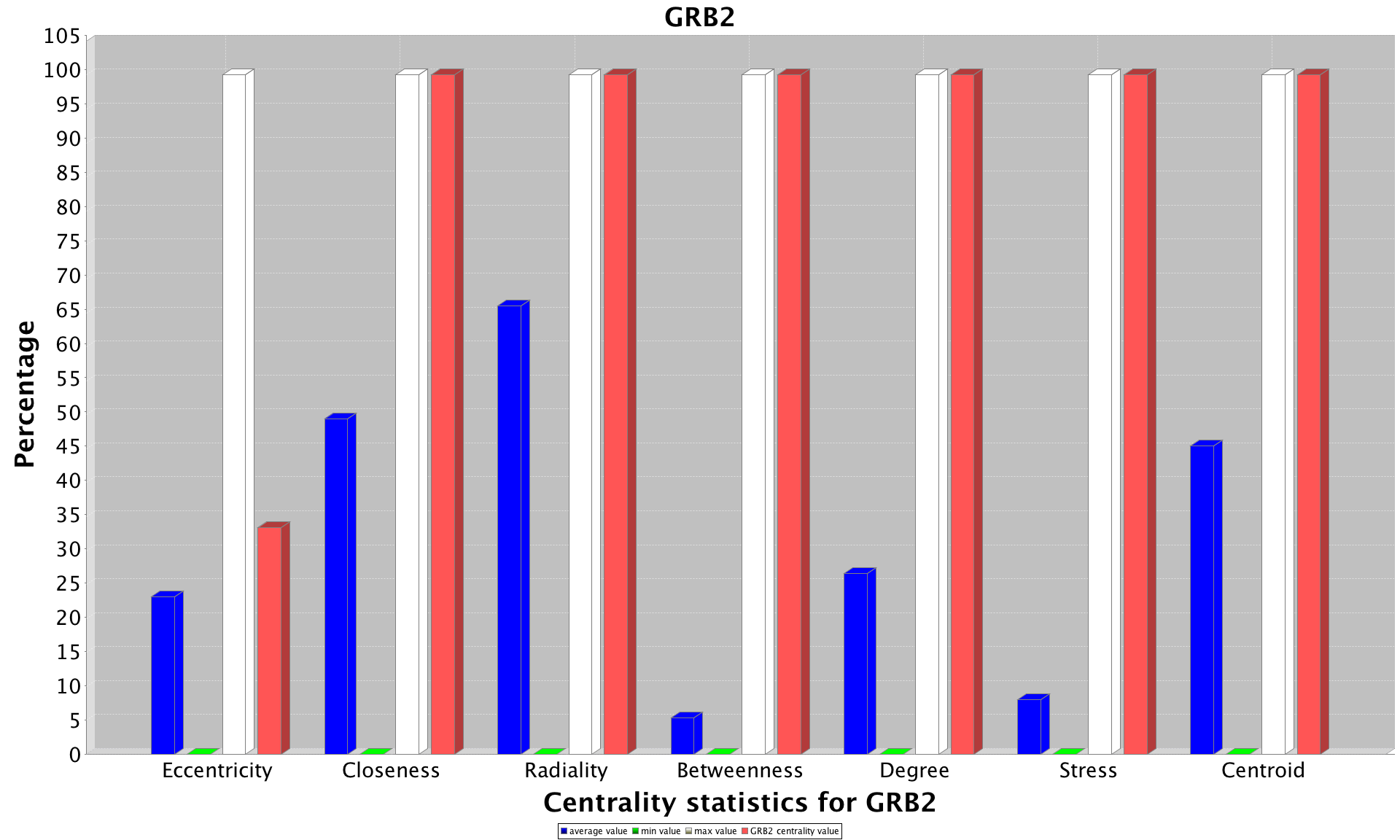

Supplement: Figure S6 — Scores of GRB2 centrality indexes in the VPS13A_to_P-tyr-network. Eccentricity, closeness, radiality, betweenness, degree, stress and centroid centrality indexes of the docking protein GRB2 in the ChAc (VPS13A_to_P-tyr)-related sub-network. The score of every index was normalized to the maximal value for every index, considered as 100%. Red columns are relative values for GRB2. Blue columns are average values. White columns are maximal values. Green columns are minimal values. (TIFF) [file pone.0031015.s006.tiff]
