# Supplementary material for: Computational Identification of Phospho-Tyrosine Sub-Networks Related to Acanthocyte Generation in Neuroacanthocytosis
Source: PLoS One. 2012 Feb 15;7(2):e31015. doi: 10.1371/journal.pone.0031015 (PMC3280254; doi:10.1371/journal.pone.0031015)
Supplement: Table S1 — List of identified proteins in comparative analysis between control and McLeod red cell membrane. (DOC) [file pone.0031015.s007.doc]

| HGNC | Protein | **Coverage**  **%** | **MLS/C** |
| --- | --- | --- | --- |
| **PLCG2** | 1-Phosphatidylinositol 4,5 bisphosphate phosphodiesterase gamma 2 | 4 | +2.1 |
| **STIP1** | Stress induced phosphoprotein protein 1 | 10 | +2.4 |
| **ACTL6A** | Actin like protein 6A | 10 | +2.7 |
| **AKT3** | Rac gamma serine-threonine kinase | 7 | +2.5 |
| **PSMC1** | Proteasome regulatory subunit 4 | 8 | +3 |
| **TPM3** | Tropomyosin alpha-3 chain | 22 | +2.6 |
| **NEU1** | Sialidase-1 precursor (G9 sialidase) | 11 | -2.07 |
| **GAPDH** | Glyceraldehyde-3 phosphate dehydrogenase | 20 | +2.4 |
| **GAPDH** | Glyceraldehyde-3 phosphate dehydrogenase | 31 | +2.9 |
| PFTK | Serine threonine protein kinase | 13 | +2.5 |
| **LRAT** | Lecithin Retinol acetyltransferase | 11 | + 2 |
| **PDAP1** | 28 KDa heat and acid stable phosphoprotein | 17 | + 2.5 |
| **ANK1** | Ankyrin-1 | 6 | +2 |
| **SPTB** | Spectrin beta | 43 | +2.3 |
| **GAPDH** | Glyceraldehyde-3 phosphate dehydrogenase | 12 | +2.4 |
| **GAPDH** | Glyceraldehyde-3 phosphate dehydrogenase | 28 | +2.9 |
| **MPP1** | Protein p55 | 9 | +2.8 |
| **EPB41** | Band 4.1 | 10 | +2.3 |
| **EPB41** | Band 4.1 | 9 | +2.7 |
| **SLC1A2** | Sodium dependent glutamate/aspartate transport 2 | 8 | -2 |
| **CUL5** | Cullin-5 | 5 | +2.4 |
| **PLD2** | Phospholipase D2 | 7 | +2.5 |
| SYK | Syk | 7 | +2.8 |
| **ICK** | Serine-threonine protein kinase ICK | 4 | +2.5 |
| **RHOBTB1** | Rho-related BTB-domain containing protein 1 | 6 | +2 |
| **MARK1** | Serine-threonine protein kinase MARK1 | 4 | +2 |
| **APEH** | Acylamino-acid releasing enzyme | 11 | +2 |
| **ACBD5** | Acyl CoA binding domain containing protein 5 | 6 | +2.4 |

HGCN:Hugo gene Nomenclature Coommitee database; MLS: McLeod syndrome
