# Supplementary material for: Computational Identification of Phospho-Tyrosine Sub-Networks Related to Acanthocyte Generation in Neuroacanthocytosis
Source: PLoS One. 2012 Feb 15;7(2):e31015. doi: 10.1371/journal.pone.0031015 (PMC3280254; doi:10.1371/journal.pone.0031015)
Supplement: Table S2 — List of identified proteins displaying different degrees of tyrosine phosphorylation in control and chorea-acanthocytosis red cell membrane. (DOC) [file pone.0031015.s008.doc]

| **HGNC** | **Protein** | **Coverage %** | **PY ChAc/C** |
| --- | --- | --- | --- |
| **SPTB** | Beta spectrin | 50 | +2.4 |
| **ANK1** | Ankyrin | 22 | +2.6 |
| **SPTB** | Beta I spectrin form beta I sigma 3 | 20 | +2.4 |
| **SLC8A3** | Sodium/calcium exchanger 3 | 7 | +3.7 |
| **SLC4A1** | Band 3 anion transport protein | 24 | +2.8 |
| **PLK3** | Serine threonine protein kinase PLK3 | 8 | +2.6 |
| **EPB41** | Band 4.1 | 24 | +4.3 |
| **EPB42** | Band 4.2 | 12 | +3.8 |
| **PPP3CC** | Serine/threonine-protein phosphatase 2B catalytic subunit gamma isoform | 11 | +2.2 |
| MPP1 | 55 kDa erythrocyte membrane protein | 34 | +2.5 |
| MPP1 | 55 kDa erythrocyte membrane protein | 18 | +2.6 |
| **ALDH1A3** | Aldehyde dehydrogenase family 1 member A3 | 6 | +2.6 |
| CAT | Catalase | 9 | +2.5 |
| PFTK | Serine threonine protein kinase PFTAIRE-1 | 10 | +2.4 |
| CAMK1 | Calcium calmodulin dependent protein kinase type 1 (CaM-K) | 10 | +2.3 |
| ACTB | Beta actin | 34 | +2.5 |
| AURKB | Serine threonine protein kinase 12 | 15 | +2.6 |
| GAPDH | Glyceraldehyde-3-P-dehydrogenase | 36 | +2.9 |
| CA2 | Carbonic Anhydrase 2 | 11 | +2.4 |
| RAB3C | Ras related protein Rab-3C | 17 | +2.7 |
| **PIP4K2A** | Phosphatidylinositol-5-phosphate 4-kinase type-2 alpha | 4 | +2.9 |
| **EPB49** | Dematin | 14 | +2.8 |
| **CELSR1** | Cadherin EGF LAG seven-pass G-type receptor 1 | 12 | +2.5 |
| **NRAS** | GTPase NRas | 14 | +2.9 |
| **RAB37** | Ras-related protein Rab-37 | 9 | +2.4 |

HGCN:Hugo gene Nomenclature Coommitee database; PY: Tyr-phosphorylation; ChAc: chorea-acanthocytosis
