# Supplementary material for: Computational Identification of Phospho-Tyrosine Sub-Networks Related to Acanthocyte Generation in Neuroacanthocytosis
Source: PLoS One. 2012 Feb 15;7(2):e31015. doi: 10.1371/journal.pone.0031015 (PMC3280254; doi:10.1371/journal.pone.0031015)
Supplement: Table S3 — List of identified proteins displaying different degrees of tyrosine phosphorylation in control and McLeod red cell membrane. (DOC) [file pone.0031015.s009.doc]

| **HGNC** | **Protein name** | **Coverage**  **(%)** | **PY MLS/C** |
| --- | --- | --- | --- |
| **GPRASP2** | G protein-coupled receptor associated sorting protein 2 | 9 | -2,69 |
| **EPB41** | protein 4.1 | 23 | -2,31 |
| **CDCP1** | CUB domain containing protein 1 | 4 | -3,93 |
| **DCXR** | L-xylulose reductase | 4 | +3,38 |
| **INMT** | indolethylamine N-methyltransferase | 9 | +3,34 |
| **GAPDH** | Glyceraldehyde-3 phosphate dehydrogenase | 31 | +2,65 |
| **POR** | NADPH cytochrone P450 reductase | 8 | -2,53 |
| **ACBD4** | acetyl-CoA binding domain containing protein 4 | 8 | +2,42 |
| **EVI5L** | **EVI5-like protein** | 4 | -2,37 |
| **GIMAP4** | GTPase IMAP family member 4 | 3 | +2,34 |
| **INMT** | indolethylamine N methyltransferase | 9 | -2,24 |
| **ANKRD23** | ankyrin repeat doain containing protein 23 | 10 | +2,19 |
| **PRKACA** | cAMP-dependent kinase catalytic subunit 3 | 5 | +2,1 |
| **ANKFN1** | ankyrin repeat and fibronectin type III domain containing protein 1 | 7 | -2,08 |
| **INMT** | indolethylammine N methyltransferase | 9 | -5,29 |
| **TTC9B** | tetratricopeptide repeat protein 9b | 10 | +4,81 |
| **ANXA4** | annexin A4 | 3 | -4,81 |
| **TTC9B** | tetratricopeptide repeat protein 9b | 10 | +4,04 |
| **PRDX6** | peroxiredoxin 6 | 9 | -3,94 |
| **INMT** | indolethylammine N methyltransferase | 9 | -3,69 |
| **NDUFB9** | NADH dehydrogenase [ubiquitinase] 1β subcomplex | 7 | +3,46 |
| **C2orf71** | uncharacterised protein C2 - C2orf71 | 3 | +3,04 |
| **ABL2** | tyrosine kinase ABL2 | 3 | +2,84 |
| **C1GALT1C1** | C1GALT1-specific chaperone 1 | 3 | +2,81 |
| **PRPH** | peripherin | 5 | -2,44 |
| **CRYBA2** | β-crystalline A2 | 9 | +2,34 |
| **CHML** | Rab protein geranylgeranyltransferasecomponent A2 | 3 | +2,21 |
| **DUSP13** | dual specificity protein phosphatase 13 | 15 | +2,17 |
| **PTGDR** | prostaglandin D2 receptor | 4 | +2,17 |
| **MYL2** | myosin light chain 2 | 7 | +2,04 |
| **RPH3AL** | Rab effector NOC2 | 9 | +2,03 |
| **MARK1** | Serine threonine protein kinase MARK1 | 4 | +2,84 |

HGCN:Hugo gene Nomenclature Coommitee database ; PY: Tyr-phosphorylation; MLS: McLeod Syndrome
